# Open fenestration discectomy versus microscopic fenestration discectomy for lumbar disc herniation: a randomized controlled trial

**DOI:** 10.1186/s12891-020-03396-x

**Published:** 2020-06-15

**Authors:** Sherwan A. Hamawandi, Injam Ibrahim Sulaiman, Ameer Kadhim Al-Humairi

**Affiliations:** 1 Department of Orthopaedics, College of Medicine, Hawler Medical University, Erbil, Iraq; 2 Department of Neurosurgery, College of Medicine, Hawler Medical University, Erbil, Iraq; 3 Dept. of Community Medicine, College of Medicine, University of Babylon, Hilla, Iraq

**Keywords:** Back pain, Leg pain, Lumbar disc herniation, Open fenestration discectomy, Microdiscectomy, Oswestry disability index, Visual analogue scale

## Abstract

**Background:**

Fenestration discectomy, for symptomatic lumbar disc herniation, is the most common surgical procedure in spine surgery. It can be done by open or microscopic procedures. This study compared the results of fenestration microdiscectomy with open fenestration discectomy in the treatment of symptomatic lumbar disc herniation as a relation to the functional outcome, leg pain, back pain, hospital stay, returns to daily activity, cost, recurrence, reoperation and type of surgery for recurrent disc herniation.

**Methods:**

60 patients age (29 - 50 years), with L4-L5 disc herniation, are divided randomly into group A- 30 patients underwent an open fenestration discectomy- and group B- 30 patients underwent fenestration microdiscectomy. All patients are assessed at 1 week, 3 months, 6 months, 12 months after surgery for Oswestry disability index and Visual analogue scale for back pain and leg pain and followed up for 4 years.

**Results:**

In both groups, all patients have minimal disability by Oswestry Disability Index after surgery.

There were significant differences between means of post-operative Visual Analogue Scale for back pain between these two groups after 1 week (3.7 in group A versus 2.2 in group B) (t = 13.28, P = < 0.001*) and after 3 months (1.73 in group A versus 0.43 in group B) (t = 10.54, P = < 0.001*).

There were no significant differences between two groups regarding post-operative VAS for leg pain, recurrence (5 patients in group A versus 4 patients in group B) and reoperation rate (2 patients in each group).

There were significant differences between means of length of hospital stay (2.10 in group A versus 1.06 in group B) (*P* < 0.001), time of returning to daily activities (7.33 in group A versus 4.03 in group B) (P < 0.001) and cost of surgery (1996.66 in group A versus 3003.3 in group B) (P < 0.001).

**Conclusion:**

Use of microscope in fenestration discectomy for treatment of symptomatic lumbar disc herniation can achieve the same goals of open fenestration regarding nerve root decompression and relief of leg pain with advantage of less back pain, less hospital staying and early return to daily activities with disadvantage of more cost with the use of microscope. With 4 years follow up, there was no significant deference in rate of recurrence and reoperation with the use of microscope but we found that type of surgery for recurrent cases may be less invasive if microscope was used in primary surgery.

**Trial registration:**

NCT, NCT04112485*. Registered 30 September 2019 - Retrospectively registered, https://clinicaltrials.gov/*NCT04112485

## Background

Lumbar disc herniation, one of the most important causes of low back pain which is one of the most common problems in orthopedics and neurosurgery, can be presented with low back pain, leg pain (radicular pain) or both. Treatment of lumbar disc herniation varies from conservative treatment, with different modalities, to surgical treatment which involved several surgical procedures starting from most invasive techniques to minimal invasive techniques [1–4].

Minimal invasive techniques for lumbar disc herniation should give the same galls of standard techniques but with minimal soft tissue damage, less blood loss and early recovery of the patient. These minimal invasive techniques for surgical treatment of lumbar disc herniation involved using microscope or endoscopy with many modifications aiming to minimize soft tissue damage and improve the early patient recovery with optimum results [5–7].

There are several studies compared between open and microscopic methods as surgical treatment of lumbar disc herniation with variable results, no clear clinical and statistical evidences showed which is superior and there is no randomized control trial study which can give clear evidence whether which method can results in less recurrent disc herniation and less reoperation rate as well as the cost of surgery and the type of surgery that may be needed for recurrent disc herniation [8–17].

Our study compared the results of open fenestration discectomy and microscopic fenestration discectomy for symptomatic lumbar disc herniation L4-L5 with follow up of 4 years in consideration of improvement in function and relief of back pain and leg pain, post-operative hospital stay, return to daily activities, complications, cost of surgery, recurrence rate of disc herniation, re-operation rate and type of surgery for recurrent disc herniation.

## Methods

### Study design and patients

#### Study design

This study was a single center, prospective, randomized, comparative, controlled trial. This study was done in Tertiary spine center hospital by neurosurgeon and orthopedic surgeon as one team from March 2015 until October 2016 and all patient were followed up for 4 year, until November 2019.

#### Patients

Sixty patients age (29-50 years) are involved in this study. Patients are divided randomly into two groups: Group A: 30 patients were treated with open fenestration discectomy, Group B: 30 patients were treated with fenestration microdiscectomy.

All the patients were suffered from symptomatic lumbar disc herniation L4-5 (which is one of the commonest site for lumbar disc herniation and we took only those patients with this level disc herniation in order to avoid bias related to different levels, so in our study only this level was included). Each patient was assessed clinically and MRI was done for all the patients to prove the presence of disc herniation and determine its level. Conservative treatment for 6 weeks was failed in all of the patients who were selected for this study.

### Method of randomization

The current study included 60 patients, the researcher put all patients in excel sheet and randomization done by computer which divided the patients into two groups (A and B); The odd numbers were put in group A and the even numbers were put in group B and the operation was done for both groups without any selection bias for the patients and the researcher follows each group for outcomes and complications.

#### Inclusion criteria

Inclusion criteria involve patients with symptomatic L4-L5 disc herniation who had failed to conservative treatment for 6 weeks.

#### Exclusion criteria

Exclusion criteria involve smoking, previous lumbar spine surgery, Diabetes Mellitus (DM), neuromuscular disorders and motor neurological deficits.

#### Follow up and outcome measures

All patients were assessed for primary outcome measure of Oswestry disability index and secondary outcome measures involving visual analogue scale for back pain and leg pain, length of hospital stay after surgery, time to return to daily activities, cost of surgery, complications (as infection), recurrence of disc herniation, rate of reoperation and type of surgery for recurrent disc herniation for a period of 4 years follow up.

All patients were assessed by Visual Analog Scale (VAS) for back pain and leg pain and Oswestry Disability Index (ODI) preoperatively and post-operatively at 1 week, 3 months, 6 months and 12 months.

No patient was lost in our study although one patient in group B travelled for studying to another country in the 2nd year of follow up and we continued with this patient through email and WhatsApp.

### Intervention

A/ Open fenestration discectomy, Group A: Under general anesthesia or spinal anesthesia with the patient in prone position, the level of L4-L5 was determined with a needle marker and fluoroscopy. A 5 cm midline incision was done, then the deep fascia was incised and the paraspinal muscles, on the symptomatic side, were retracted to expose the lamina. Fenestration was done in the lamina and removal of ligamentum flavum was done to decompress the nerve root and by using the nerve retractor, the herniated disc was identified and excised in case of sequestrated disc herniation while in case of contained disc herniation, a small oblique opening was done by a tenotome through the herniated part of the disc and by pituitary rongeur the herniated part of the disc was removed. Hemostasis was secured and the incision was closed in layers without drain.

B/ Fenestration microdiscectomy, Group B: Under general or spinal anesthesia with the patient in prone position, the operating level was identified by a needle marker and fluoroscopy. A midline skin incision of 1.5 cm was done. The deep fascia was incised and the paraspinal muscles were retracted on the symptomatic side using Casper reactor to expose the lamina of L4 then with a diamond high speed drill, the inferior part of lower lamina of L4 was drilled to enable of passing a hook under the ligamantum flavum which was incised by a tenotome over the underneath hook then by a Kerrison, part of ligament flavum was removed to expose the nerve root and by nerve root retractor the herniated disc was exposed and removed with a rongeur. In cased of contained disc herniation, a small oblique opening was done by a tenotome and the herniated part of the disc was removed by pituitary rongeur. Hemostasis was secured by bipolar electrocautery and incision closed in layers without drain.

#### Postoperative care

Early patient’s mobilization was done as pain allowed and stitches were removed 2 weeks after surgery.

#### Postoperative complications

Fortunately, we have no complications like dural tear or discitis except one patient in group A got superficial infection and was treated successfully by oral antibiotics and daily dressing.

### Data analysis

Statistical analysis was carried out using SPSS version 21 (SPSS, IBM Company, Chicago, USA). Categorical variables were presented as frequencies and percentages. Continuous variables were presented as (Means ± SD). Student t-test was used to compare means between two groups. Paired t-test was used to compare means for paired reading. Pearson’s chi square (X^2^) was used to find the association between categorical variables. A *p-*value of ≤0.05 was considered as significant.

## Results

### Demographic data

In current study, the mean age of patients was (41.35 ± 6.50) years. Younger patient was 29 years old and the older patient was 50 years old. Male represents (36.7%) from the sample, female represents (63.3%) of the sample. There were no significant differences between means of age between two study groups ((group A (41.26 ± 6.45) and group B (41.43 ± 6.66)). (t = − 0.098, *P* = 0.922).

Regarding gender, the percentage of both male and female are equal between two groups; 11 males (36.7%) and 19 females (63.3%).

### Primary outcome measure: (Oswestry disability index)

**Fig. 1:**
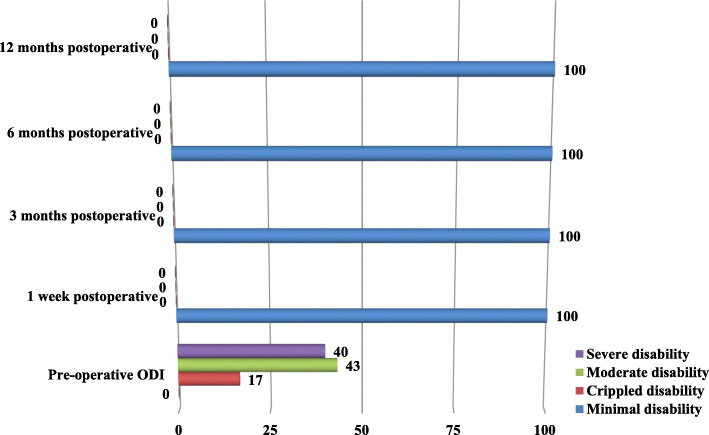
The distribution of group A patients according to ODI pre-operatively and 1 week, 3 months, 6 months and 12 months postoperatively

The distribution of group A patients, according to ODI pre-operatively and 1 week, 3 months, 6 months and 12 months postoperatively, was shown in Fig. 1. Before surgery, 40% of patients had severe disability, 43% of patients had moderate disability and 17% of patients had crippled disability, while after operations all patients had minimal disability in 4 postoperative periods of assessments.

**Fig. 2:**
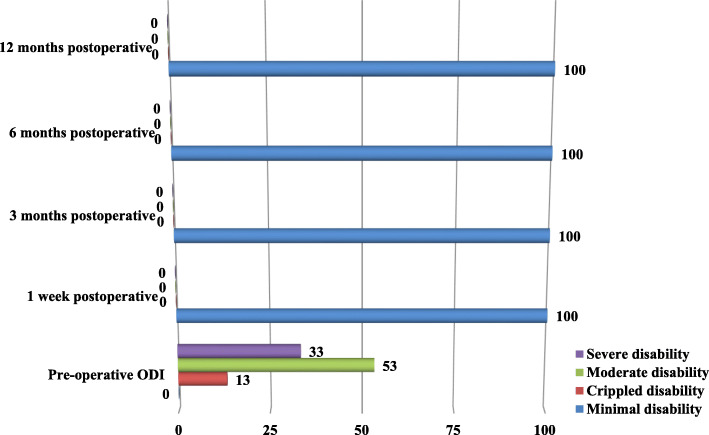
The distribution of group B patients according to ODI pre-operatively and 1 week, 3 months, 6 months and 12 months postoperatively

The distribution of group B patients, according to ODI pre-operatively and 1 week, 3 months, 6 months and 12 months postoperatively, was shown in Fig. 2.Before surgery, 33% of patients had severe disability, 53% of patients had moderate disability and 13% of patients had crippled disability, while after surgery all patients had minimal disability in 4 postoperative periods of assessments.

### Secondary outcome measures

#### A/ VAS for back pain between group a and group B

**Fig. 3:**
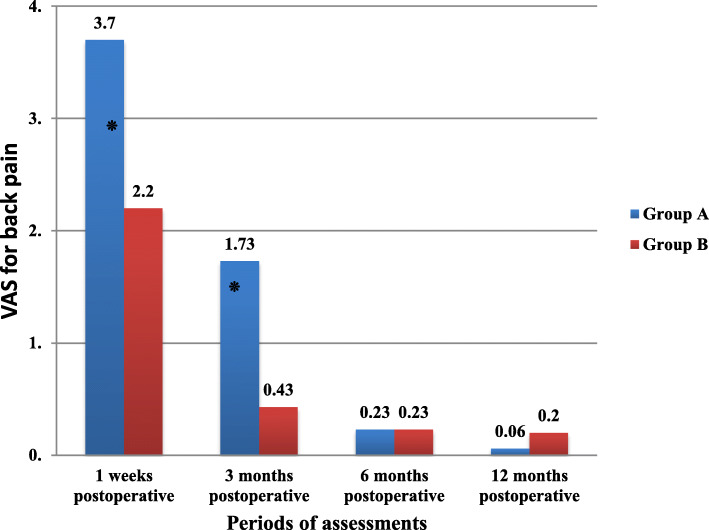
The mean differences of post-operative VAS for back pain between study groups

The mean differences of post-operative VAS for back pain between study groups, including (group A and group B) in four periods of assessments, were shown in Fig. 3. There were significant differences between means of post-operative VAS for back pain between these two groups after 1 week (3.7 in group A versus 2.2 in group B) (t = 13.28, P = < 0.001*) and after 3 months (1.73 in group A versus 0.43 in group B) (t = 10.54, P = < 0.001*), while non-significant differences between two groups after 6 months of operation (0.23 in group A versus 0.23 in group B) (t = 0.00, *P* = 1.000) and 12 months of operation (0.06 in group A versus 0.2 in group B) (t = − 1.523.00, *P* = 0.134).

#### B/ VAS for leg pain between group a and group B

**Fig. 4:**
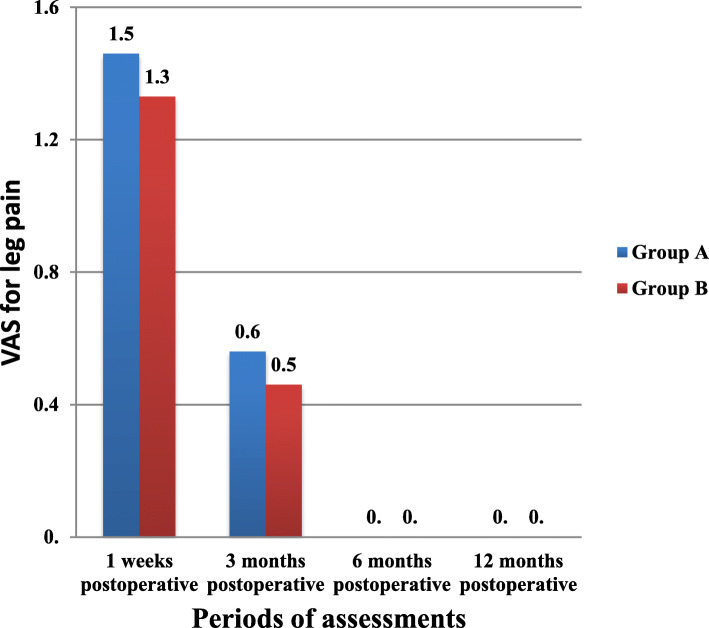
The mean differences of post-operative VAS for leg pain between study groups

The mean differences of post-operative VAS for leg pain between study groups, including (group A and Group B) in four periods of assessments, were shown in Fig. 4. There were no significant differences between means of post-operative VAS for leg pain between these two groups after 1 week (1.5 in group A versus 1.3 in group B) (t = 1.046, *P* = 0.3) and after 3 months(0.6 in group A versus 0.5 in group B) (t = 0.766, *P* = 0.447).

#### C/ VAS for back pain in group a in different periods (multiple comparison)

The mean differences of VAS for back pain between pre-operative and post-operative assessments in four time periods (after 1 weeks, 3 months, 6 months and 12 months), for **group A patients,** were shown in Table 1. There were significant differences between means of VAS for back pain in pre-operative assessment (7.73) and post-operative assessments in four time periods (3.7, 1.73, 0.23 and 0.06 respectively) (*P* < 0.001).

**Table 1:** The mean differences of (VAS for back pain) between pre-operative and post-operative assessments in four time periods

Study variables	Periods of assessment	N	Mean	SD	***P***-value
VAS for back pain	VAS for back pain preoperatively	30	7.73	0.78	< 0.001*
	VAS for back pain 1 weeks postoperatively	30	3.70	0.46	
	VAS for back pain 3 months postoperatively	30	1.73	0.44	
	VAS for back pain 6 months postoperatively	30	0.23	0.43	
	VAS for back pain 12 months postoperatively	30	0.06	0.25	

Friedman Test, *P* ≤ 0.05 was significant

#### D/ VAS for back pain in group B in different periods (multiple comparison)

The mean differences of VAS for back pain between pre-operative and post-operative assessments in four time periods (after 1 weeks, 3 months, 6 months and 12 months), for **group B patients,** were shown in Table 2.There were significant differences between means of VAS for back pain in pre-operative assessment (7.66) and post-operative assessments in four time periods (2.2, 0.43, 0,23 and 0.2 respectively) (*P* < 0.001).

**Table 2:** The mean differences of (VAS for back pain) between pre-operative and post-operative assessments in four time periods

Study variables	Periods of assessment	N	Mean	SD	***P***-value
VAS for back pain	VAS for back pain preoperatively	30	7.66	0.75	< 0.001*
	VAS for back pain 1 weeks postoperatively	30	2.20	0.40	
	VAS for back pain 3 months postoperatively	30	0.43	0.50	
	VAS for back pain 6 months postoperatively	30	0.23	0.43	
	VAS for back pain 12 months postoperatively	30	0.20	0.40	

Friedman Test, *P* ≤ 0.05 was significant

#### E/ VAS for leg pain in group a in different periods (multiple comparison)

The mean differences of VAS for leg pain between pre-operative and post-operative assessments in four time periods (after 1 weeks, 3 months, 6 months and 12 months), for **group A patients,** were shown in Table 3.There were significant differences between means of VAS for leg pain in pre-operative assessment (9.63) and post-operative assessments in four time periods (1.46, 0.56,0.0 and 0.0 respectively) (*P* < 0.001).

**Table 3:** The mean differences of (VAS for leg pain) between pre-operative and post-operative assessments in four time periods

Study variables	Periods of assessment	N	Mean	SD	***P***-value
VAS for leg pain	VAS for leg pain preoperatively	30	9.63	0.49	< 0.001*
	VAS for leg pain 1 weeks postoperatively	30	1.46	0.50	
	VAS for leg pain 3 months postoperatively	30	0.56	0.50	
	VAS for leg pain 6 months postoperatively	30	0.00	0.000	
	VAS for leg pain 12 months postoperatively	30	0.00	0.000	

Friedman Test, *P* ≤ 0.05 was significant

#### F/ VAS for leg pain in group B in different periods (multiple comparison)

The mean differences of VAS for leg pain between pre-operative and post-operative assessments in four time periods (after 1 weeks, 3 months, 6 months and 12 months), for **group B patients,** were shown in Table 4. There were significant differences between means of VAS for back pain in pre-operative assessment (9.53) and post-operative assessments in four time periods (1.33, 0.46, 0.0 and 0.0 respectively) (*P* < 0.001).

**Table 4:** The mean differences of (VAS for leg pain) between pre-operative and post-operative assessments in four time periods

Study variables	Periods of assessment	N	Mean	SD	***P***-value
VAS for back pain	VAS for back pain preoperatively	30	9.53	0.50	< 0.001*
	VAS for back pain 1 weeks postoperatively	30	1.33	0.47	
	VAS for back pain 3 months postoperatively	30	0.46	0.50	
	VAS for back pain 6 months postoperatively	30	0.00	0.00	
	VAS for back pain 12 months postoperatively	30	0.00	0.00	

#### C/ hospital stay, time of returning to daily activities and cost of surgery

The mean differences of length of hospital stay, time of returning to daily activity and cost of surgery between study groups including (group A and Group B) were shown in Table 5. There were significant differences between means of length of hospital stay (2.10 in group A versus 1.06 in group B) (*P* < 0.001), time of returning to daily activities (7.33 in group A versus 4.03 in group B) (P < 0.001) and cost of surgery (1996.66 in group A versus 3003.3 in group B) (P < 0.001).

**Table 5:** The mean differences of length of hospital stay, time of returning to daily activities and the cost of surgery between study groups

Study variables	Study group	N	Mean	SD	t-test	***P***-value
**Length of hospital stay (days)**	Group A	30	2.10	0.30	**14.26**	< 0.001*
	Group B	30	1.06	0.25		
**Time of returning to daily activity (days)**	Group A	30	7.33	0.84	**14.73**	< 0.001*
	Group B	30	4.03	0.88		
**Cost ($)**	Group A	30	1996.66	39.24	**−85.95**	< 0.001*
	Group B	30	3003.33	50.74		

#### C/ post-operative complications

One patient, in group A, got superficial wound infection and was treated successfully by oral antibiotics and daily dressing.

#### D/ rate and time of the recurrence of disc herniation during 4 years of follow up

The association between study group including (group A and group B) and the recurrence of disc herniation were shown in Table 6. There was no significant association between study group and the rate of recurrence of disc herniation (5 patients in group A versus 4 patients in group B) (*P* = 1.000).

**Table 6:** Association between study group and the recurrence

Study variables	Study group		Total	***P***-value	Odds	95% CI
	Group A	Group B				
**Recurrence**				1.000	1.30	0.313-5.404
Yes	5 (16.7)	4 (13.3)	9 (15.0)			
No	25 (83.3)	26 (86.7)	51 (85.0)			
Total	30 (100.0)	30 (100.0)	200 (100.0)			

*P value ≤0.05 was significant

The mean differences of time of the recurrence between study groups including (group A and Group B) were shown in Table 7. There were no significant differences in means of time of the recurrence between study groups (29 months after operation in group A versus 27 months after operation in group B) (*P* = 0.778)

#### E/ level of disability by ODI at time of recurrence of disc herniation during 4 years of follow up

**Fig. 5:**
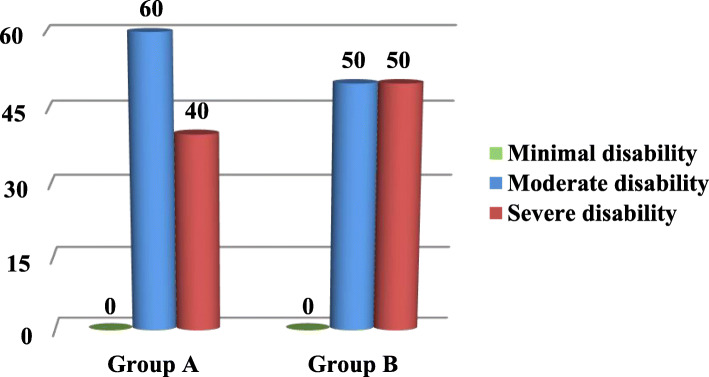
Association between study group and type of disability at time of the recurrence (*P* = 1.000)

The association between study group and type of disability on recurrence of disc herniation was shown in Fig. 5. There was no significant difference in the level of disability between the two groups at time of the recurrence. In group A, 2 patients had severe disability and 3 patients had moderate disability, while in group B, 2 patients had severe disability and 2 patients had moderate disability) (*P* = 1.000).

#### E/ reoperation rate and type of surgery for recurrent disc herniation within 4 years of follow up

**Fig. 6:**
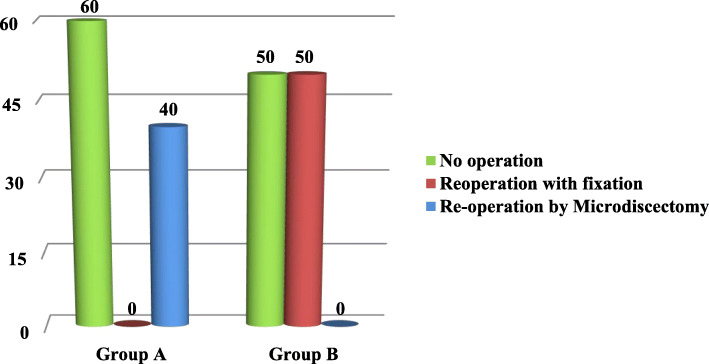
Association between study group and intervention at time of the recurrence (*P* = 0.365)

The reoperation rate and type of intervention for the recurrent cases between the two groups were shown in Fig. 6. There were 5 recurrent cases in group A; 3 of them were treated by conservative treatment while 2 patients were treated by surgical decompression and posterior stabilization with pedicle screws, while in group B, there were 4 recurrent cases; 2 of them were treated by conservative treatment and other 2 patients were treated by microdiscectomy. There was no significant difference between two groups (*P* = 0.365).

## Discussion

### Regarding Oswestry disability index

When we compare the ODI preoperatively and postoperatively through all periods of assessment in both groups A and B, there is significant deference which means that both methods of treatment are effective in achieving excellent functional improvement for patients with symptomatic lumbar disc herniation. (Figs. 1 and 2),

### Regarding visual analogue scale for back pain

There were significant differences between means of post-operative VAS for back pain between these two groups after 1 week (t = 13.28, P = < 0.001*) and after 3 months (t = 10.54, P = < 0.001*), while non-significant differences between two groups after 6 months (t = 0.00, *P* = 1.000) and 12 months of operation (t = − 1.523.00, *P* = 0.134) (Fig. 3). This can explain how fenestration microdiscectomy is minimal invasive technique with less tissue damage than open fenestration discectomy so the back pain was less in group B in early stages due to less interference with soft and bony tissues.

When we compare VAS for back pain in each group between preoperative and 4 periods of assessments (multiple comparison), we found there were significant differences between means of VAS for back pain pre-operative and post-operative assessments in four time periods (Tables 1 and 2) and this explained by that fenestration discectomy, whether open or microscopic, can remove disc herniated fragments so the cause of local irritation was removed.

### Regarding visual analogue scale for leg pain

There were no significant differences in means of post-operative VAS for leg pain between these two groups after 1 week (t = 1.046, *P* = 0.3) and after 3 months (t = 0.766, *P* = 0.447) as shown in Fig. 4. This will explain that both techniques (open and microdiscectomy) are effective in decompression of the nerve root and removal the herniated disc fragments.

When we compare VAS for back pain in each group between preoperative and postoperative 4 periods of assessments (multiple comparison), we found that there were significant differences in means of VAS for leg pain between pre-operative and post-operative assessments in four time periods (Tables 3 and 4) and this can be explained by that fenestration discectomy, whether open or microscopic, can remove disc herniated fragments so the cause of nerve root irritation was removed.

### Regarding hospital stay, return to daily activities and cost of surgery

There were significant differences in means of length of hospital stay, time of returning to daily activities and cost of surgery between these two groups (Table 5). In group B, because of minimal tissue damage in fenestration microdiscectomy and less back pain postoperatively so the patients can be discharged home early and can return to daily activities sooner. The cost of use of the microscope made the cost of surgery in group B significantly more than in group A.

### Regarding post-operative complications

Fortunately, we didn’t get dural tear or discitis but we got superficial wound infection in one patient of group A and was treated successfully with daily dressing and oral antibiotics.

### Regarding rate and time of the recurrence of disc herniation with period of 4 years of follow up

There were 5 cases of recurrent disc herniation in group A and 4 cases of recurrence in group B (Table 6) and the cause of recurrence in all patients was lifting heavy weight. This difference in number of recurrent cases was not statistically significant (*P* = 1.000) as well as the time of recurrence between the groups was not statistically significant (Table 7) (*P* = 0.778).

**Table 7:** The mean differences in means of time of the recurrence between study groups

Study variables	Study group	N	Mean	SD	t-test	***P***-value
**Time of recurrence (months)**	Group A	5	29.00	6.08		0.778
	Group B	4	27.75	6.70		

### Regarding level of disability by ODI at time of recurrent disc herniation with period of 4 years of follow up

There was no significant difference in level of disability by ODI at time of the recurrence of disc herniation between both groups (Fig. 5) (P = 1.000).

### Regarding re-operation rate and type of intervention for recurrent disc herniation with period of 4 years of follow up

Two patients from the 5 recurrent cases in group A underwent reoperation for recurrent disc herniation after failure to respond to conservative treatment and two patients from 4 recurrent cases in group B underwent reoperation after failure of conservative treatment. There was no significant difference between two groups but the type of reoperation was different between two groups; in group A decompression and posterior stabilization by pedicle screws was needed while in group B reoperation was done without need for posterior stabilization and this may be explained by that in group B minimal bony removal for discectomy can decrease the risk of destabilization of vertebral segment so in second surgery there is less need for fixation (Fig. 6) (*P* = 0.365).

### Comparing our study with other studies

1/ Henriksen et al. [18]; A controlled study of microsurgical versus standard lumbar discectomy.

**Table:** 

Henriksen et al. study	Our study
Microscopic procedure does not shorten the length of a stay at the hospital.	There is significant decrease in length of hospital stay with microscopic procedure. (*P* < 0.001)

2/ Tureyen K [19]; One-level one-sided lumbar disc surgery with and without microscopic assistance

**Table:** 

Study of Tureyen K	Our study
The length of postoperative inpatient stay was 1 day in both groups. (*p* > 0.05)	There is significant decrease in length of hospital stay with microscopic procedure. (P < 0.001)
Patients in the microsurgery-treated group returned to work in less time. (*p* < 0.001)	There is significant less time needed to return to daily activities with microscopic procedure. (*P* < 0.001)

3/ Katayama et al. [20]; Comparison of surgical outcomes between macro discectomy and micro discectomy for lumbar disc herniation:

**Table:** 

Katayama et al. study	Our study
Statistically significant differences were observed in duration of hospitalization with microscopic procedure.	There is significant decrease in length of hospital stay with microscopic procedure. (P < 0.001)
Statistically significant differences were observed in postoperative VAS for lumbar pain with microscopic procedure.	There were significant differences between means of post-operative VAS for back pain between these two groups after 1 week (t = 13.28, P = < 0.001*) and after 3 months of operation (t = 10.54, P = < 0.001*), while non-significant differences between two groups after 6 months (t = 0.00, P = 1.000) andtwelve months of operation. (t = −1.523.00, P = 0.134).

4/ Porchet et al. [21]; Microdiscectomy compared with standard discectomy

**Table:** 

Porchet et al. study	Our study
There were no group differences in duration of hospital stay. (*P* > 0.05)	There is significant decrease in length of hospital stay with microscopic procedure. (P < 0.001)
There was no clinically relevant difference in outcome after lumbar disc excision dependent on the use of the microscope.	There is significant less time needed to return to daily activities with microscopic procedure. (P < 0.001)

We noticed that there was no randomized control trial compared between open and microscopic fenestration discectomy in regarding cost of surgery, the recurrence of disc herniation, reoperation rate and type of intervention for recurrent cases with period of follow up for 4 year or more and this may give our study its importance.

## Conclusion

Use of microscope in fenestration discectomy for treatment of symptomatic lumbar disc herniation can achieve the same goals of open fenestration regarding nerve root decompression and relief of leg pain with advantage of less back pain, less hospital staying and early return to daily activities with disadvantage of more cost with the use of microscope. In our study with 4 years follow up there was no significant deference in rate of recurrence and reoperation with the use of microscope but we found that type of surgery for recurrent cases may be less invasive if microscope was used in primary surgery.

### Limitations of our study and our recommendations

1/ Limited number of patients involved in this study.

2/ We recommend further randomized controlled trials with larger sample size with longer period of follow up and other levels of disc herniation than L4-L5 can be involved.

## Data Availability

The datasets used and analyzed during the current study are available from the corresponding author on reasonable request.

## Abbreviations

MRI: Magnetic resonance imaging
L: Lumbar
DM: Diabetes Mellitus
VAS: Visual analogue scale
ODI: Oswestry Disability Index
cm: Centimeter
